# Drug Resistance Driven by Cancer Stem Cells and Their Niche

**DOI:** 10.3390/ijms18122574

**Published:** 2017-12-01

**Authors:** Marta Prieto-Vila, Ryou-u Takahashi, Wataru Usuba, Isaku Kohama, Takahiro Ochiya

**Affiliations:** Division of Molecular and Cellular Medicine, National Cancer Center Research Institute, Tokyo 104-0045, Japan; mprietov@ncc.go.jp (M.P.-V.); rytakaha@ncc.go.jp (R.T.); wusuba@ncc.go.jp (W.U.); ikohama@ncc.go.jp (I.K.)

**Keywords:** cancer stem cells, drug resistance, cancer niche

## Abstract

Drug resistance represents one of the greatest challenges in cancer treatment. Cancer stem cells (CSCs), a subset of cells within the tumor with the potential for self-renewal, differentiation and tumorigenicity, are thought to be the major cause of cancer therapy failure due to their considerable chemo- and radioresistance, resulting in tumor recurrence and eventually metastasis. CSCs are situated in a specialized microenvironment termed the niche, mainly composed of fibroblasts and endothelial, mesenchymal and immune cells, which also play pivotal roles in drug resistance. These neighboring cells promote the molecular signaling pathways required for CSC maintenance and survival and also trigger endogenous drug resistance in CSCs. In addition, tumor niche components such as the extracellular matrix also physically shelter CSCs from therapeutic agents. Interestingly, CSCs contribute directly to the niche in a bilateral feedback loop manner. Here, we review the recent advances in the study of CSCs, the niche and especially their collective contribution to resistance, since increasingly studies suggest that this interaction should be considered as a target for therapeutic strategies.

## 1. Cancer Stem Cells

### 1.1. Introduction

Cancer is one of the most devastating diseases worldwide. In 2016, more than 1.5 million people were afflicted in the U.S. Despite recent major advances in understanding the molecular and genetic basis of cancer, more than one third of afflicted people die each year [1]. The leading cause of treatment failure is cancer cells resistance to drug treatment, which leads to tumor recurrence and metastasis. Metastasis remains the cause of more than 90% of cancer-related deaths [2].

Recent studies suggest that cancer stem cells (CSCs) are the main source of this resistance. Tumor-initiating cells or cancer stem cells, as they will be referred into this article, are a small subpopulation of cells within a tumor that retain the capacity for self-renewal and are able to differentiate into the heterogeneous lineages that comprise the tumor. They also possess high tumorigenicity capacity and are thought to be highly resistant to radiation and chemotherapy, making them capable of repopulating a tumor after treatment [3]. CSCs are localized in a specific microenvironment referred to as the niche, which is formed by a variety of cells that promote CSC survival and enhance their characteristics.

An in-depth understanding of the biological characteristics of CSCs and in particular their role in drug resistance, is crucial for establishing novel tumor diagnostic and therapeutic strategies. In this article, we review the recent known mechanisms used by CSCs to overcome drug treatment, as well as the role of the niche in CSCs and drug resistance and examine their implications for novel therapeutic strategies.

### 1.2. CSC History and Origin

The concept of CSCs was first described in 1971 by Perce and Wallance, who showed that aggressive undifferentiated cells gave rise to benign, well-differentiated cells in squamous cell carcinoma in vivo [4]. In 1997, John Dick identified a subpopulation of cells in patients with acute myeloid leukemia that was different from the bulk, these leukemic cells exhibited stem-like properties. These cells were described as cancer stem cells [5]. In the following years, CSCs were observed in many kinds of solid cancers, beginning with breast CSCs. Al-Hajj et al. used flow cytometry to identify a small subpopulation of breast cells that displayed the markers CD44^+^/CD24^−^/Lin^−^. As few as 100 cells were able to form tumors in immunocompromised mice [3]. Shortly after, CSCs were also identified in human brain tumors. Singh et al. found a subpopulation of brain tumor cells that exhibited high proliferation, self-renewal and differentiation ability; all of these cells carried the neural stem cell (SC) surface marker CD133 and were described as brain CSCs [6]. CSCs have since been identified in many other solid tumors including prostate, colon and pancreas [7,8,9,10]. CSCs share several features with normal SCs [11]; however, this does not mean that CSCs originate from SCs that become malignant. The origin of CSCs is not yet clear and the CSC state is not static. Various reports describe CSC-enriched populations giving rise to non-CSCs, while non-CSCs can also generate a CSC population [12,13,14]. CSC plasticity within a tumor appears to be dependent on context and environment, as illustrated by a study examining the plasticity of melanoma CSCs. Purified cells expressing JARID1B, a histone demethylase considered to be a melanoma CSC marker, generated JARID1B-negative cells, as expected by the CSC model; however, a single JARID1B-negative cell could also give rise to JARID1B-positive cells [15]. Thus and taking in consideration the plasticity of cancer cells, it seems fair to say that both theories could be asserted.

### 1.3. Regulators in CSC Phenotype

SCs are essential for the maintenance of tissue homeostasis within multicellular organisms. A principal characteristic of SCs is self-renewal, the biological process where, upon cell division, a SC produces one (asymmetric division) or two (symmetric division) daughter cells that retain the capacity for self-renewal. This ensures that a pool of SCs remains available [16]. In the case of asymmetric division, the other resulting cell differentiates to preserve tissue homeostasis.

Cancer stem cells are so-named because they share the capacity for long-term self-renewal with SCs. In addition to this characteristic, most of the critical SC pathways including Wnt/B-catenin, Notch and Hedgehog can be found altered in CSCs [17].

Wingless (Wnt)/β-catenin: Upregulation of Wnt/β-catenin signaling has been reported to cause dedifferentiation in both tumor and terminally differentiated cells—such as gastric epithelial cells lines—in which the overexpression of Wnt/β-catenin signaling also induced the generation of fundic gland polyps [18,19]. In head and neck squamous cell carcinoma, Wnt/β-catenin promoted the expression of the stemness markers CD44 and ALDH as well as the drug resistance markers ABCG2 and ABCC4, which signify cisplatin-induced resistance. These changes could be reversed using sFRP4, a Wnt pathway antagonist that not only downregulated drug resistance markers but also reversed the epithelial-to-mesenchymal transition (EMT), as evidenced by the downregulation of N-cadherin and the re-establishment of E-cadherin [20]. Wnt is also a very important signaling pathway for SCs but its targets are currently used as CSC markers. For example, LGR-5 was first described as a SC marker in the intestine but is currently used as a colon cancer CSC marker [21,22].

Sonic Hedgehog: Overexpression of the Hedgehog pathway has been implicated in the maintenance of self-renewal in CSCs from lung squamous cell carcinoma, glioma, colon cancer and breast cancer through regulation of the expression of OCT4, SOX2 and BMI1 [23,24,25,26,27].

Notch: In cancer, Notch signaling is deregulated and promotes self-renewal in breast CSCs [28,29,30] and in oral squamous cell carcinoma by controlling the expression of *SLUG* and *TWIST*. Crosstalk with the Wnt and Hedgehog pathways also impacts Notch signaling [31].

Telomerase reactivation: Telomere shortening causes chromosome instability, fusion and ultimately senescence [32]. The long-term self-renewal capacity of tumor cells is due in part to the activation of telomerase a reverse transcriptase enzyme that adds terminal repeats to the 3’ end of telomeres [33,34,35]. For example, one study found telomerase activated in 88–94% of invasive breast carcinoma and none of normal breast tissue [36].

### 1.4. Epithelial-to-Mesenchymal Transition and CSC Phenotype

The activation of stem-related signaling pathways such as Notch, Hedgehog and Wnt promotes EMT, the phenomenon by which carcinoma cells acquire a CSC phenotype. During this process, epithelial cells lose their polarity; change their morphology from a cobblestone-like epithelial appearance to an elongated, fibroblastic-like shape; and switch off cadherin, which involves the downregulation of E-cadherin and the upregulation of N-cadherin [37,38]. EMT plays a fundamental role in the developmental processes of mesoderm and neural tube formation, as well as wound healing [39]. Cancer cells also undergo EMT through regulators such as SNAIL, SLUG, ZEB1/2 and TWIST, which suppress E-cadherin expression binding to conserved E-box sequences (mainly CAGGTG-type) in E-cadherin promoters [40].

The relation between EMT and CSC phenotype acquisition has been extensively studied [11]. For instance, in breast cancer, the addition of Twist, Snail and FOXC2 increased the mesenchymal properties of cancer cells. Those cells gained mammosphere formation ability and the subpopulation of CD44^high^/CD24^low^—described as CSC—increased [41,42]. Similar phenomena were observed in prostate cancer cells that exhibited the EMT phenotype: Cells increased the expression of prostate CSC markers SOX2, NANOG, OCT4, LIN28B and NOTCH1 and exhibited enhanced sphere-forming ability [43].

## 2. Intrinsic Drug Resistance of CSCs

Drug resistance represents an ongoing challenge in cancer treatment and is doubtless the main reason for treatment failure [44,45]. Clinical drug resistance is characterized by resistance not only to one drug but also commonly to a broad spectrum of drugs, a phenomenon referred to as multidrug resistance (MDR) [46,47]. MDR limits the possibility of overcoming the problem using similar-but-different drugs. There are two categories of resistance: (1) Acquired resistance, which develops as a response to treatment and (2) Intrinsic resistance, in which resistance to a spectrum of drugs is present even if the drugs in question have never been used against a specific tumor [45,48].

It has been widely described that CSCs harbor endogenous resistance mechanisms against radiation and chemotherapy at a much higher rate than non-CSC, differentiated tumor cells [49]. Therefore, chemotherapy and radiotherapy treatment eliminate the bulk of the population of non-CSCs but not CSCs [50].

CSC populations expressing CD133^+^ cells in multiple cancers, including glioblastoma, non-small cell lung cancer and colon cancer, presented worse 5-year overall survival and higher rates of chemotherapy and radiation resistance than the CD133-negative cells [49,51,52]. After radiation treatment in glioblastoma, CSC populations increased 2–4-fold, probably due to a preferential activation of the DNA damage response.

**Figure 1:**
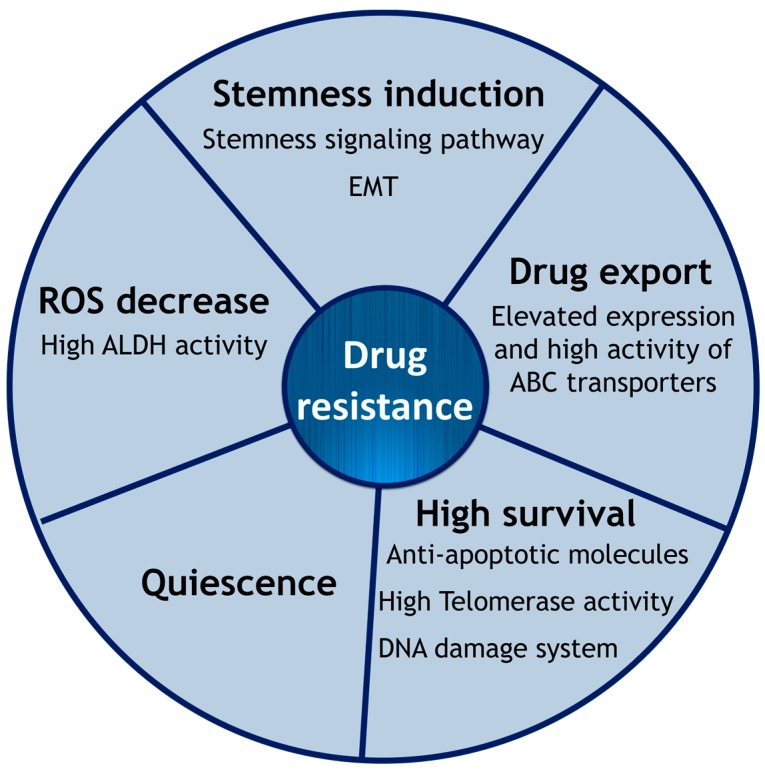
Radio- and chemotherapy resistance that CSCs intrinsically possess.

The main reason for drug resistance seems to reside in the similarities between CSCs and normal SCs [17]. Because SCs maintain the pool of cells in an organism, it is biologically essential to keep and protect these SCs. Therefore, SCs have evolved several mechanisms to avoid death by apoptosis or cell senescence. CSCs appear to utilize these mechanisms against anti-cancer therapies. The major mechanisms of MDR against commonly used therapeutic drugs such as cisplatin, paclitaxel, docetaxel and cetuximab are described below [53] (Figure 1).

### 2.1. Epithelial-to-Mesenchymal Transition

EMT activates SC signaling pathways inducing CSC characteristics that increase drug resistance. In most cases, the molecular mechanisms responsible for EMT and the resulting resistance remain uncertain. In head and neck squamous cell carcinoma, EMT has been associated with Hedgehog signaling and acquired chemoresistance [54]. Three murine mammary basal epithelial cell lines exhibited EMT induction in response to TGFβ, a well-known EMT inducer. The induction of EMT in response to TGFβ conferred resistance to UV-induced apoptosis. Interestingly, the response to TGFβ was highly dependent on the extension of cell-to-cell contact [55].

Cells that undergo EMT may enter into a state of quiescence, in which the cell is no longer dividing [56]. These cells will not be affected by most conventional treatments, which target actively dividing cells [17]. For instance, a SNAIL-mediated EMT phenotype in oral cancer cells exhibited quiescence and the cells were highly resistant to chemotherapeutics [57]. In larynx cancer, the subpopulation of cells with CD44^high^/EGFR^low^ expression exhibited EMT and quiescent phenotypes and had reduced sensitivity towards common anti-cancer drugs such as cisplatin, cetuximab and gefitinib [58]. In summary, the factors that activate EMT also promote stemness and quiescence, which may enable drug resistance in multiple cancers.

### 2.2. Cell Membrane Transporters: ABC Family

ATP-binding cassette (ABC) transporters play a crucial role in the development of MDR, due to their ability to efflux toxic chemicals from the cell [4,59]. These proteins belong to a family of 49 membrane proteins commonly involved in the transport of compounds and small molecules from the cytosol to the extracellular medium using ATP hydrolysis. CSCs express a high number of these proteins on their cell surface [60]. ABCB1 (also known as MDR1 or P-gp), ABCG2 (also known as BCRP1), ABCB5 and ABCC1 are the most well-characterized [61]. ABCG2 transporter is capable of expelling a large range of xenobiotic compounds such as topotecan, irinotecan and doxorubicin. Similarly, multiple myeloma cells with increased expression of ABCB1 in cell-surface, that was identified by the actively efflux of CDy1 dye identifying a subpopulation of CSCs, exhibited resistance to carfilzomib. ABCB1 was also found to be linked to the Hedgehog pathway, supporting the relationship between CSCs and ABC transporters [62].

On the other hand, the overexpression of ABC transporters can be used to identify CSC subpopulations within a tumor by staining a population of cells with Hoechst 33,342 dye and Rhodamine 123 dye [63,64]. These dyes are pumped out of CSCs, which can therefore be identified as the unstained subpopulation in flow cytometry [65].

### 2.3. Hypoxia and ROS

Hypoxia plays a pivotal role in the development and maintenance of self-renewal and therefore contributes to the maintenance of CSC characteristics [66]. Indeed, CSCs are usually located nearby hypoxic zones within tumors [67]. It has been reported that, in gastric cancer cells and neuroblastomas, the hypoxic microenvironments induced EMT and concretely in neuroblastoma, it elevated the expression of SC markers such as Notch1 [68,69].

The main regulator of cellular responses to hypoxia is hypoxia-inducible factor 1-alpha (HIF1α). At high oxygen levels, HIF1α is ubiquitinated and subsequently degraded. When oxygen levels decrease, ubiquitination is inhibited and HIF1α is activated, translocating into the nucleus where it dimerizes with hypoxia-inducible factor 1B (HIF1B) and activates the transcription of hypoxia response elements [70]. This includes the transcription of over 60 genes that promote angiogenesis to assist in the delivery of oxygen, as well as activating proliferation and survival pathways [71].

Hypoxia is involved in drug resistance mainly by two systems: Stem-related pathway activation and quiescence promotion. Through the activation of HIF1α, the expression of many EMT and stemness activators are induced. HIF1α induces the expression of Wnt, Hedgehog and notch pathways, as well as other stemness markers such as cMET, CD133, NANOG, SOX2, FOXA2, SOX17 and PDX1 [72,73]. At the same time, because hypoxia results in the restriction of nutrients as well as oxygen, it is an unfavorable condition for cellular growth and induces quiescence in cancer cells [11,74]. The HIF1α signaling pathway also promotes drug resistance in CSCs by decreasing the production of reactive oxygen species (ROS). Under normal conditions, ROS accumulation leads to apoptosis in both normal and cancer cells [75,76]. The low level of ROS described in CSCs is critical for preserving SC properties as well as to induce quiescence [17,77]. In mammospheres—composed almost entirely of SCs—the cells contain lower levels of ROS, which explains the increased radioresistance when compared with adherent, differentiated cells [78,79]. Likewise, oral cancer cell lines containing low levels of ROS due to the overexpression of antioxidant enzymes such as catalase SOD2 and peroxiredoxin 3 exhibited CSC properties and were cisplatin-resistant when compared with ROS^high^ cells [80].

One of the molecules responsible for ROS decrease is also a widely described CSC marker, aldehyde dehydrogenase (ALDH) [81]. ALDH is a family of 19 cytosolic enzymes involved in the oxidation of intracellular aldehydes and in the oxidation of retinol to retinoic acid during early stages of SC differentiation [82,83]. ALDH1, the main isoform, not only facilitates detoxification by directly reducing ROS but also by producing antioxidant compounds such as NADP. ALDH1 also protects cells against alkylating agents such as paclitaxel [16]. In multiple gastric cancer cells lines and hematopoietic malignances, ALDH expression levels were significantly higher in ROS-low than in ROS-high cells and most of these cells were quiescent [84,85,86]. Tumors expressing high levels of ALDH are tumorigenic and resistant to chemotherapy in cancers of colon, breast, lung, pancreas, bladder, prostate and ovary [84]. ALDH1A1 enhances activation of DNA repair in ovarian cancer cells, underscoring the importance of this enzyme as a defense against radio and chemotherapeutic agents [77].

### 2.4. High Survival Capacity of CSCs

Despite the fact that CSCs possess many mechanisms to avoid cell death, some drugs circumvent all of these barriers and succeed in damaging CSC DNA. However, CSCs have other ways to overcome this damage. CSCs of lung, pancreas, glioma and breast possess highly active DNA damage response systems [49,79]. In studies of oral cancer, the EMT factor SNAIL promoted the expression of nucleotide excision repair protein (ERCC1). This resulted in higher DNA damage repair and generated cisplatin resistance [87].

In addition to DNA repair systems, CSCs avoid apoptosis through the mutation or inactivation of cell cycle-regulating genes as well as apoptotic inducing genes [88]. One of the most well-known proteins regulating the apoptotic pathway is p53 and its isoforms p63 and p73. Loss of p53 function in colon, breast and lung carcinoma promoted EMT through Snail expression, resulting in increased radioresistance [88]. In many cases, treatment with chemotherapy such as cisplatin induced apoptosis resistance. Cisplatin pretreatment in cells contributed to the interaction of HDAC and TRIB1, which is overexpressed in leukemia and prostate cancer and together inactivated p53 [89].

CD133^+^ glioma CSCs contributed to radioresistance through an enhanced cell cycle checkpoint response, resulting in DNA repair due to the overexpression of the cell cycle checkpoint, NBS1. This was further confirmed after the inhibition of other checkpoint kinases, Chk1 and Chk2, re-sensitized the cells [49].

### 2.5. Effect of MicroRNAs in CSC Phenotype Acquisition

Epigenetic factors are also known to play an important role in drug resistance acquisition. One of these epigenetic factors are microRNAs. MicroRNAs are short (20–22 nucleotide), non-coding RNA, that regulate the expression of genes by binding the 3’ untranslated region (UTR) and repressing the target mRNA translation [90]. MicroRNAs have been described to participate in cancer progression, malignance, drug resistance and even in CSCs properties acquisition [91,92]. One exemplification is the microRNA-22, which increases the hematopoietic stem cells self-renewal though the inactivation of TET2 in myelodysplastic syndrome and leukemia, where was found upregulated [93]. Furthermore, the same microRNA was shown to induce EMT through TET-micro-RNA200 axis and to promote the metastasis in breast cancer mouse model [94].

As described previously, EMT activation promotes stem-like phenotype and consequent drug resistance [54]. Likewise, microRNAs involved in EMT activation have also been described to promote CSCs phenotype and drug resistance. The loss of microRNA-200 family increased the expression of EMT related genes, such as ZEB1, ZEB2, Snail and Slug in pancreatic CSCs both in vitro and in vivo [92].

Hypoxia also promotes changes in microRNA expression profiles, such as the inhibition of microRNA-34a expression, which in normal circumstances downregulates stem cell-regulatory genes and inhibits the CSC phenotype [91]. Under hypoxia, the up-regulation of Slug decreased microRNA-34a, which in turn increased the expression of the hypoxia-induced tumor carbonic anhydrase iso-enzyme 9 (CA9). The induction of this gene eventually promoted the survival of breast CSCs [95,96]. Importantly the authors identified the active secretion of microRNA-34a within exosomes, membrane vesicles approximately 100 nm in diameter and essential for cell-to-cell communications [96,97], indicating a role of microRNA in the surrounding environment.

Hypoxia also induced microRNA-21 in pancreatic cells leading to the acquisition of EMT phenotype and allowing them to escape apoptosis [98].

Other microRNAs also have been described to be related with drug-resistance, one example is the microRNA-27b. Loss of microRNA-27b was found to increase docetaxel resistance in Luminal A breast-type cancer by increasing the expression and membrane location of ABCG2. It also promoted CSC phenotype [99].

All these reports suggest that epigenetic factors such as microRNAs participates in multiple pathways promoting CSCs phenotypes and consequent drug resistance.

## 3. Tumor Heterogeneity and Cancer Niche

### 3.1. Tumor Heterogeneity

Drug resistance is the major reason for therapy failure in cancer patients and there is an increased awareness that tumor heterogeneity is a critical reason for this treatment failure [45,100]. Intratumoral heterogeneity, which was first observed over 60 years ago, refers to the observation that tumors are composed of multiple morphologically and phenotypically distinct subpopulations of cancer cells [101]. Indeed, massively parallel sequencing studies show that spatial and temporal heterogeneity are phenomena that occur commonly in tumors [102]. Two main models explain the origin of cancer heterogeneity: The stochastic or Darwinian model and the CSC model.

Stochastic model: This model is based on the postulate of Charles Darwin as described in his book “The Origin of Species”, in which the fittest organism is best suited to survive. The cells within the tumor are considered an ecosystem in which spontaneous mutations and epigenetic changes may confer cells with greater fitness for a specific tumor microenvironment; likewise, the fittest clone will repopulate the tumor after a treatment. The tumor may contain multiple subclones that are evolving independently, resulting in a dominant genetic clone and many more genetically distinct subclones [45].

Cancer Stem Cell model: This hypothesis attributes heterogeneity to aberrant differentiation programs of CSCs and presupposes the existence of hierarchical organization of cancer cells, resembling the SC hierarchy in tissues [103]. On the top of the hierarchy, there is a small subpopulation of cells with self-renewal and differentiation capacity that give rise to differentiated, phenotypically diverse cancer cells, which compose most of the tumor and possess much lower tumorigenicity.

Currently, it is commonly accepted that these models are not mutually exclusive and it is believed that both contribute to intratumor heterogeneity. In this mixed theory, CSCs are at the top of the hierarchy and give rise to other cells that make up the tumor but are still susceptible to sporadic mutations and environmental factors that select the fittest clones [103]. 

### 3.2. Cancer Niche

SC maintenance and regulation is crucial for the organism’s survival, as well as for the prevention of uncontrolled cell growth [103]. SC division and differentiation occur in specialized microenvironments referred to as the niche. The niche environment regulates SCs through cell-cell communication, both between SCs and with differentiated cells, as well as through cell-ECM communication, paracrine communication, hormonal signaling, growth factors, cytokines and physiochemical factors such as oxygen levels [17,104].

CSCs are also thought to reside in niches that consist of numerous types of stromal cells, including endothelial cells, mesenchymal cells, immune cells and fibroblasts [105,106]. The CSC niche also includes factors secreted by these cells, such as growth factors or cytokines [11,107] and the extracellular matrix.

The CSC niche may promote an imbalance between CSC self-renewal and differentiation, leading to the proliferation of tumor cells, invasion and metastasis. The CSC niche also plays an important role in therapy response [108].

### 3.3. Cancer Niche and Therapy Resistance

**Figure 2:**
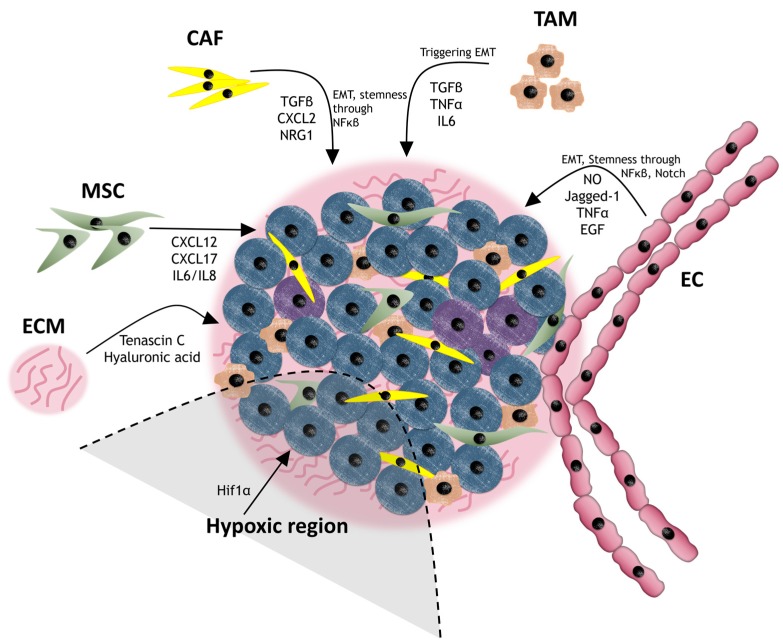
CSCs (represented as purple cells) within the cancer niche, surrounded by CAFs, TAMs, ECs, MSCs and ECM. The CSCs are situated in a hypoxic region and are receiving stimuli from neighboring cells that increase drug resistance.

The tumor microenvironment supports initiation, growth, migration and metastasis and also takes part in therapeutic resistance, largely by the support of stem-related signaling pathway maintenance in CSCs [21] (Figure 2 and Table 1).

#### 3.3.1. Cancer-Associated Fibroblasts (CAFs)

Fibroblasts are the most abundant component of tumor stroma and therefore niche, in many cancers. This is especially true in breast and pancreatic cancer [117,129]. Fibroblasts found in tumors are called cancer-associated fibroblasts (CAFs) and share many similarities with fibroblasts that actively participate in wound healing at inflammatory sites [130,131]. Compared with normal fibroblasts, CAFs have increased proliferation, enhanced production of ECM components and unique cytokine secretion, including SDF-1, CXCL12, VEGF, PDGF and HGF [132,133].

The main component secreted by CAFs is TGFβ, which induces EMT and ultimately drug resistance [133]. The high level of secretion of TGFβ into the medium by a cell line derived from invasive breast tumors has been reported to induce EMT in those cells [109]. CAFs also promoted the stemness of CSCs in other types of cancer, including gastric and breast. In particular, CAF promoted stemness by the secretion of NRG1 and activating the NF-κB signaling in gastric cancer [110]. Tamoxifen resistance was increased 4.4-fold in breast cancer cell lines as a result CAF co-culture [134]. 

CAF-derived exosomes increased drug resistance of colon CSCs to 5-fluorouracil by activating Wnt signaling [113]. Similar studies showed that exosomes derived from CAF activated STAT1 in breast cancer cells through the receptor RIG-1; at the same time STAT1 activation further activated NOTCH3, which increased drug resistance in CSCs [114].

Gastric, breast, prostate and glioma cells increased their self-renewal capacity in response to NRG1 secreted by CAF, which activated NF-κB signaling [111,112] inducing CSCs properties. It is important to note that CAF not only induced drug resistance by promoting stemness signaling pathways in CSCs but also by secreting type I collagen, which contributes to decreasing drug uptake [115].

#### 3.3.2. Mesenchymal Stem Cells (MSCs)

Mesenchymal stem cells (MSCs) are adult SCs with the ability to differentiate into a variety of skeletal tissue cells. Under normal conditions, MSCs act as immunomodulators; however, when located in the stroma, they induce CSC phenotype by activating the NF-κB pathway through the secretion of multiple cytokines and chemokines such as CXCL12, CXCL7 and IL6/IL8 [17]. These secretions also promote treatment resistance in breast cancer [116]. Additionally, the physical interaction between MSCs and breast cancer activates the non-receptor tyrosine kinase Src and its downstream PI3K/Akt pathway and enhances resistance to trastuzumab [117]. Interaction with MSCs also increased resistance of epithelial ovarian cancers to carboplatin and paclitaxel, mediated by the acquisition of MDR proteins [118].

#### 3.3.3. Endothelial Cells (ECs)

Blood vessels are lined with endothelial cells (ECs). This vasculature plays a key role in the tumor microenvironment because it supplies the tumor with oxygen and nutrients. In 2007, it was first suggested that the vascular microenvironments support the maintenance of a self-renewing CSC pool in brain tumors [135]. Later, it was confirmed that ECs secreted several growth factors, including EGF, that induced EMT, promoting the maintenance of CSC properties in glioblastoma and colorectal and head and neck squamous cell carcinoma [119,120]. In head and neck squamous cell carcinoma, it has been reported that 80% of the CSCs are located close to blood vessels [136].

Recently, ECs have been reported to enhance properties of CSCs in glioma and colorectal cancer by Notch signal activation through a nitric oxide signaling pathway and the soluble form of Jagged-1, respectively [121,122]. In breast cancer, it was reported that ECs secreted TNFα, which activated a NF-κB signaling pathway in CSC and induced the secretion of several factors, including CXCL1/2, which attracted myeloid cells into the tumor and produced chemokines including S100A8/9, which eventually generated chemoresistance to doxorubicin and cyclophosphamide. Furthermore, CXCR2 blockers were able to reduce the resistance [112]. An additional way in which ECs protect CSCs from therapeutic drugs is by the irregular shape of tumor blood vessels, which diminishes the ability of drugs to reach CSCs [123].

#### 3.3.4. Tumor-Associated Macrophages (TAMs)

The contributions of cellular components of inflammation, such as monocytes and macrophages recruited into tumor tissue, have been extensively studied [137]. Macrophages are derived from CD34^+^ bone marrow progenitors, which can be found in the bloodstream as monocytes. After extravasation into tissues, they differentiate into a tissue-specific macrophage [138]. In the tumor microenvironment, macrophages are regarded as tumor-associated macrophages (TAMs) or M2 and interact with cancer cells through a wide range of growth factors, cytokines and chemokines.

TAMs induce drug resistance in non-small cell lung cancer though the secretion of a multitude of factors, such as TGFβ and TNFα, which are potent EMT stimulators [124,125]. Activation of the STAT3 pathway, partly through IL6, resulted in trastuzumab resistance and an increase in HER2-positive cells in breast CSCs [126]. Similarly, it boosted CSCs properties in the pancreas.

#### 3.3.5. Extracellular Matrix (ECM)

The extracellular matrix (ECM) is a collection of molecules mostly secreted by fibroblasts. The ECM plays a critical role in the tumor microenvironment, since in order to form a tumor, cancer cells must form attachments to the ECM. In solid tumors, increased ECM stiffness is a physical barrier separating therapeutics from the cells and thus protecting CSCs from chemotherapeutic agents [139]. Moreover, the ECM contains several proteins that interact with membrane proteins in CSCs and activate stem and proliferative signaling pathways, as well as drug resistance. For instance, hyaluronic acid, which is abundant in the ECM, is the CD44 receptor’s ligand. Upon interaction, it mediates the acquisition and maintenance of CSC properties [127]. In addition, tenascin C enhances the efficiency of the signaling pathways of Wnt and Notch, stabilizing breast CSCs [128].

## 4. Contribution of CSCs to the Niche

**Figure 3:**
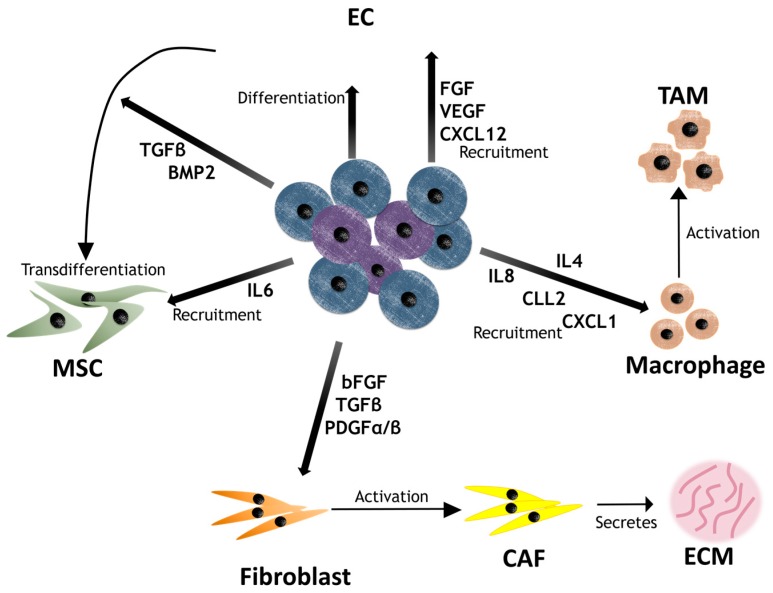
Factors secreted by CSCs (represented as purple cells) promote the recruitment and activation of niche components, indicating that the relation of CSC-niche is not unidirectional.

As mentioned above, the tumor microenvironment not only supplies growth-promoting signals that induce the generation and maintenance of CSCs but it also takes part in therapeutic resistance. Subsequent studies suggest that CSCs also promote the recruitment of niche components as well as contribute directly to the microenvironment through differentiation [11]. Understanding this bidirectional crosstalk between CSCs and their niche is critical to understanding and overcoming therapeutic resistance. Below, we analyze the contribution of CSCs to the CSC niche (Figure 3).

CAFs: CSCs manipulate neighboring fibroblasts into cancer-associated cells (CAFs) by the secretion of several factors, including PDGF-α/β b-FGF, IL6 and TGFβ [117,140,141,142]. In breast cancer cells, these cytokines activated STAT3, inducing their activation and further CCL2 expression in CAF that resulted in CSCs activating Notch1 signaling and promoting the maintenance of stemness [143]. Valenti et al. described how breast CSCs also secreted Hedgehog ligand, activating the Hedgehog signaling pathway in CAFs, which promoted the secretion of factors that enable CSC maintenance. The Hedgehog inhibitor vismodegib reduced the number of CAFs, breaking the positive feedback loop and reducing tumor progression [144].

MSCs: Breast CSCs recruit MSCs by secreting IL6, which induces CXCL7 production in MSCs. CXCL7 supports tumor growth and drug resistance in mouse model [133]. Furthermore, several studies have demonstrated that CSCs can differentiate into functional ECs [145,146]. ECs can transdifferentiate into MSCs under BMP2 or TGFβ stimulation, suggesting that MSCs can be generated from CSCs [147].

ECs: In a manner similar to that observed for MSCs, CSCs are able to both recruit ECs and differentiate directly into them. Cancer cells induce angiogenesis by secreting factors such as Hif1α, VEGFA, CXCL12 and FGF [148]. Additional studies have shown evidence that these factors were mainly secreted by the CSC subpopulation [49]. Glioma CD133^+^ CSCs secreted 10-fold more VEGF than the CD133^−^ subpopulation in both hypoxia and normoxia [49,149]. In other studies, CSCs were found to differentiate into functional ECs under environmental changes such as hypoxia or glucose depravation [150]. The resulting cells expressed the EC markers CD31, CD34 and vWF. Similar phenomena were observed when breast and renal spheres were cultured with serum and VEGF, acquiring endothelial markers and the ability to organize into capillary-like structures [115,151]. One of the most significant findings regarding the contribution of CSCs to the cancerous niche is the direct differentiation of glioblastoma CD133^+^ CSCs into vascular functional ECs expressing CD31. These cells lined functional vessels after being injected into nude mice [148].

TAMs: CSCs are able to recruit macrophages into the tumor by producing pro-inflammatory cytokines and chemokines. The expression of RAS oncoproteins in cancer cells promotes the secretion of IL6, IL8 and CXCL1, which recruit macrophages [152,153]. Once inside the tumor, the macrophages are activated by factors such as IL4 and transformed into TAMs. IL4 also plays an essential role in wound healing. Other cytokines such as CCL2 promote TAM infiltration in both primary and metastatic regions in breast and skin squamous cell carcinoma [154]. Moreover, an antagonist of the CCL5 receptor—maraviroc—inhibited TAM recruitment in a mouse model [155].

## 5. Treatments and Approaches

CSCs are thought to be the cells that remain after treatment due to enhanced chemo- and radioresistance, resulting in the subsequent recurrence of tumors. Therefore, to eliminate the possibility of tumor relapse, therapy should focus on the identification and complete eradication of the CSC population [4,156,157].

Due to the lack of specific markers, many studies have tried to eradicate CSCs by targeting signaling pathways highly activated in these cells. One example of a relatively satisfactory therapy for basal cell carcinoma is vismodegib, an inhibitor of the Hedgehog pathway. In phase II clinical trials, vismodegib treatment resulted in an increased median survival of one year in comparison with patients receiving a standard treatment [158,159]. These results suggested that vismodegib decreased the CSCs population through Hedgehog inhibition, being a promising strategy to the usage of vismodegib as a combinatory treatment with, for example, radiotherapy to target both non-CSC and CSC to remove all tumor cells [160,161].

Another example for CSC-targeting therapies in vitro and xenografted human cancers is salinomycin, a polyether ionophore antibiotic that is isolated from *Streptomyces albus*. It is thought that salinomycin interferes with ABC drug transporters and inhibits Wnt/β-catenin signaling pathway [162]. Currently, synthetic derivatives of salinomycin are being generated to improve the understanding of its elusive mechanism of action; one example is ironomycin, which was found to induce cell death by accumulating and sequestering iron in the lysosomes [163]. Importantly, the ability of salinomycin to kill both CSCs and therapy-resistant cancer cells contains a reasonable therapeutic potential in combination with other drugs in all stages of human cancer.

To improve the aforementioned gaps in current research, many clinical trials have been trying a combination of conventional treatments, which target actively dividing cells, along with adjuvant therapies that specifically target CSCs. These adjuvant therapies may either target CSC stem-related pathways or increase the sensitivity of CSC in multiple ways. One interesting strategy is CSC differentiation induction, which, in some hematopoietic malignancies, improved the prognosis of patients notably. For instance, all-*trans* retinoic acid (ATRA) induced terminal differentiation of leukemic promyelocytes, leading to apoptotic death. Since 2009, ATRA is a component of APL treatment [164]. Recently, ATRA properties have been squeezed and its efficiency has been proved in other solid tumors such as breast cancer. Yan et al. demonstrated that radioresistant MCF7 cell line, after ATRA treatment, induced its differentiation re-sensitizing them to epirubincin treatment [165]. Following this approach, differentiation induction strategies by exploiting the capacity of CSCs to differentiate could be very powerful.

Another strategy is to reduce CSC resistance, sensitizing them to traditional therapies. For this approach, the most used drug for type II diabetes, metformin, is a promising candidate [166]. Metformin was shown to reduce the breast CSC subpopulation, partially through the inhibition of an ABC transporter located in the cell membrane [99,167]. Currently, metformin is under clinical trials as an adjuvant therapy [168].

Another promising drug is doxycycline. Doxycycline, a FDA-approved antibiotic, is a drug that also has been described to reduce bone metastasis from breast cancer and reduce tumor burden in pancreas [169,170]. Marianna de Francesco et al. showed that CSC present a strict dependence on mitochondrial biogenesis for its proliferation and survival and that doxycycline was capable to inhibit this mitochondrial biogenesis and reduce the CSCs fraction resistant to Paclitaxel [171]. Currently, clinical studies are being conducted in advanced breast cancer [NCT01847976].

As mentioned above, CSCs have high expression levels of ALDH, which decreases the levels of ROS, protecting the cells from the toxic effects of DNA damage by ROS and subsequent apoptosis [172]. Previous studies showed that simultaneous knockdown of two ALDH isoforms results in increased cyclophosphamide sensitivity of lung cancer cells, suggesting the possible utility of ALDH-targeting treatments [173].

As we have described in this review, CSCs possess intrinsic resistance that is enhanced by their niche. Thus, besides directly targeting CSCs, simultaneously attacking their microenvironment is a very promising novel strategy. Recent findings demonstrate that CSCs can be newly generated from differentiated non-CSCs by reprogramming mechanisms such as EMT [11]. Blockade of EMT could be accomplished by targeting the components of the tumor microenvironment such as tumor-associated CAFs or TAMs that secrete factors that induce EMT [109,124,174]. Pro-tumorigenic factors supplied by innate immune cells during chronic inflammation could be another key factor in, for example, colon cancer, where inflammation is a critical factor. Clinical studies have demonstrated that long-term use of anti-inflammatory anti-cyclooxygenase-2 (COX-2) reduces the risk of colon cancer by 40–50% [175]. Another COX-2 inhibitor, celecobix, also showed the reduction of colorectal CSC subpopulation [176]. A similar treatment is being applied in colitis-associated colon cancer (CAC).

The ECM and its associated proteins are other promising targets. In an animal model of pancreatic cancer, stroma reduction through the enzymatic destruction of hyaluronic acid led to reduced interstitial pressure, re-expanding the vasculature and enabling increased delivery of standard chemotherapy with concomitant increased efficacy [177], suggesting that not only cancer cells or CSCs are possible anticancer targets.

## 6. Conclusions

Traditional therapies against the bulk of cancer cells are not sufficient to eradicate all cells within the tumor, especially those that exhibit high resistance to treatment, such as CSCs. However, treating only these CSCs does not succeed in tumor eradication either. 

We have need to keep in mind that CSCs are surrounded by a complex group of cells, referred to as the CSC niche, which secretes multiple factors promoting not only CSC survival but also plasticity and drug resistance. Because the CSC niche is essential for CSC survival and drug resistance, targeting these niche components is a promising strategy for achieving better treatment outcomes. Multiple studies have proposed potential novel targets for drug therapies, in hopes of eradicating MDR.

## Figures and Tables

**Table 1:** Molecules secreted by cancer niche components that promote drug resistance in CSCs.

Component	Molecule	Effect	Cancer	Ref.
CAF	TGFβ secretion	EMT stimulator	Gastric, prostate	[109]
	NRG1 secretion	Activation of NF-κB signaling pathway	Gastric, breast, prostate, glioma	[110,111,112]
	CAF exosomes	Activation of Wnt signaling pathway	Colon	[113,114]
	Collagen type I secretion	Decrease of drug uptake	-	[115]
MSC	CXCL12, CXCL7, IL6/IL8 secretion	Activation of NF-κB signaling	Breast	[17,116]
	Physical interaction	Activation of SCR and its downstream PI3K/Akt	Breast	[117]
	Physical interaction	Increase of MDR protein expression	Ovarian	[118]
EC	TNFα secretion	Recruitment of myeloid cells that induce the loop CXCL1/2-S100A8/9	Breast	[112]
	EGF secretion	EMT stimulator	Glioblastoma, colorectal, HNSCC	[119,120]
	Nitric oxygen, Jagged-1 secretion	Activation of Notch signaling pathway	Glioma, colorectal	[121,122]
	-	Direct blockage of drug administration due to irregular vessel shape	-	[123]
TAM	TGFβ and TNFα secretion	EMT stimulator	NSCLC	[124,125]
	IL6 secretion	Activation of STAT3	Breast, pancreas	[126]
ECM	ECM stiffness	Physical barrier that physically separates the cells	-	[125]
	Hyaluronic acid secretion	Activation of CD44	Breast	[127]
	Tenascin C secretion	Activation of Wnt and Notch signaling pathways	Breast	[128]

## Abbreviations

CSC	Cancer stem cell
SC	Stem cell
Wnt	Wingless
EMT	Epithelial-to-mesenchymal transition
ROS	Reactive oxygen species
ECM	Extracellular matrix
CAF	Cancer-associated fibroblast
MSC	Mesenchymal stem cell
EC	Endothelial cell
TAM	Tumor-associated macrophage

## References

[1] Siegel R.L., Miller K.D., Jemal A. (2016). Cancer statistics. CA Cancer J. Clin..

[2] Lawson D.A., Bhakta N.R., Kessenbrock K., Prummel K.D., Takai K., Zhou A., Eyob H., Balakrishnan S., Wang C., Yaswen P. (2015). Single-cell analysis reveals a stem-cell program in human metastatic breast cancer cells. Nature.

[3] Al-Hajj M., Wicha M.S., Benito-Hernandez A., Morrison S.J., Clarke M.F. (2003). Prospective identification of tumorigenic breast cancer cells. Proc. Natl. Acad. Sci. USA.

[4] Lobo N.A., Shimono Y., Qian D., Clarke M.F. (2007). The biology of cancer stem cells. Annu. Rev. Cell Dev. Biol..

[5] Lapidot T., Sirard C., Vormoor J., Murdoch B., Hoang T., Caceres-Cortes J., Minden M., Paterson B., Caligiuri M.A., Dick J.E. (1994). A cell initiating human acute myeloid leukaemia after transplantation into SCID mice. Nature.

[6] Singh S.K., Hawkins C., Clarke I.D., Squire J.A., Bayani J., Hide T., Henkelman R.M., Cusimano M.D., Dirks P.B. (2004). Identification of human brain tumour initiating cells. Nature.

[7] Patrawala L., Calhoun T., Schneider-Broussard R., Li H., Bhatia B., Tang S., Reilly J.G., Chandra D., Zhou J., Claypool K. (2006). Highly purified CD44+ prostate cancer cells from xenograft human tumors are enriched in tumorigenic and metastatic progenitor cells. Oncogene.

[8] Hurt E.M., Kawasaki B.T., Klarmann G.J., Thomas S.B., Farrar W.L. (2008). CD44+ CD24(−) prostate cells are early cancer progenitor/stem cells that provide a model for patients with poor prognosis. Br. J. Cancer.

[9] Ricci-Vitiani L., Lombardi D.G., Pilozzi E., Biffoni M., Todaro M., Peschle C., de Maria R. (2007). Identification and expansion of human colon-cancer-initiating cells. Nature.

[10] Li C., Heidt D.G., Dalerba P., Burant C.F., Zhang L., Adsay V., Wicha M., Clarke M.F., Simeone D.M. (2007). Identification of pancreatic cancer stem cells. Cancer Res..

[11] Eun K., Ham S.W., Kim H. (2017). Cancer stem cells heterogeneity: Origin and new perspectives on CSC targeting. BMB Rep..

[12] Magee J.A., Piskounova E., Morrison S.J. (2012). Cancer Stem Cells: Impact, Heterogeneity, and Uncertainty. Cancer Cell.

[13] Gupta P.B., Fillmore C.M., Jiang G., Shapira S.D., Tao K., Kuperwasser C., Lander E.S. (2011). Stochastic state transitions give rise to phenotypic equilibrium in populations of cancer cells. Cell.

[14] Brooks M.D., Burness M.L., Wicha M.S. (2015). Therapeutic Implications of Cellular Heterogeneity and Plasticity in Breast Cancer. Cell Stem Cell.

[15] Roesch A., Fukunaga-kalabis M., Schmidt E.C., Susan E., Brafford P.A., Vultur A., Basu D., Gimotty P., Vogt T., Herlyn M. (2010). A temporarily distinct subpopulation of slow-cycling melanoma cells is required for continuous tumor growth. Cell.

[16] Takahashi K., Yamanaka S. (2006). Induction of Pluripotent Stem Cells from Mouse Embryonic and Adult Fibroblast Cultures by Defined Factors. Cell.

[17] Carnero A., Garcia-Mayea Y., Mir C., Lorente J., Rubio I.T., LLeonart M.E. (2016). The cancer stem-cell signaling network and resistance to therapy. Cancer Treat. Rev..

[18] Scheel C., Eaton E.N., Li S.H., Chaffer C.L., Reinhardt F., Kah K.J., Bell G., Guo W., Rubin J., Richardson A.L. (2011). Paracrine and autocrine signals induce and maintain mesenchymal and stem cell states in the breast. Cell.

[19] Radulescu S., Ridgway R.A., Cordero J., Athineos D., Salgueiro P., Poulsom R., Neumann J., Jung A., Patel S., Woodgett J. (2013). Acute WNT signalling activation perturbs differentiation within the adult stomach and rapidly leads to tumour formation. Oncogene.

[20] Warrier S., Bhuvanalakshmi G., Arfuso F., Rajan G., Millward M., Dharmarajan A. (2014). Cancer stem-like cells from head and neck cancers are chemosensitized by the Wnt antagonist, sFRP4, by inducing apoptosis, decreasing stemness, drug resistance and epithelial to mesenchymal transition. Cancer Gene Ther..

[21] Barker N., van Es J.H., Kuipers J., Kujala P., van den Born M., Cozijnsen M., Haegebarth A., Korving J., Begthel H., Peters P.J. (2007). Identification of stem cells in small intestine and colon by marker gene Lgr5. Nature.

[22] Takahashi H., Ishii H., Nishida N., Takemasa I., Mizushima T., Ikeda M., Yokobori T., Mimori K., Yamamoto H., Sekimoto M. (2011). Significance of Lgr5+ve cancer stem cells in the colon and rectum. Ann. Surg. Oncol..

[23] Hoffmann W. (2013). Self-renewal of the gastric epithelium from stem and progenitor cells. Front. Biosci. (Schol. Ed.).

[24] Cochrane C.R., Szczepny A., Watkins D.N., Cain J.E. (2015). Hedgehog signaling in the maintenance of cancer stem cells. Cancers.

[25] Justilien V., Walsh M.P., Ali S.A., Thompson E.A., Murray N.R., Flieds A.P. (2014). The PRKCI and SOX2 oncogenes are co-amplified and cooperate to activate hedgehog signaling in lung squamous cell carcinoma. Cancer Cell.

[26] Molofsky A.V., Pardal R., Iwashita T., Park I., Clarke M.F., Morrison S.J. (2003). Bmi-1 dependence distinguishes neural stem cell self-renewal from progenitor proliferation. Nature.

[27] Muñoz P., Iliou M.S., Esteller M. (2012). Epigenetic alterations involved in cancer stem cell reprogramming. Mol. Oncol..

[28] Androutsellis-Theotokis A., Leker R.R., Soldner F., Hoeppner D.J., Ravin R., Poser S.W., Rueger M.A., Bae S.-K., Kittappa R., McKay R.D.G. (2006). Notch signalling regulates stem cell numbers in vitro and in vivo. Nature.

[29] Chiba S. (2006). Concise review: Notch signaling in stem cell systems. Stem Cells.

[30] Driessens G., Beck B., Caauwe A., Simons B.D., Blanpain C. (2012). Defining the mode of tumour growth by clonal analysis. Nature.

[31] Takebe N., Warren R.Q., Ivy S.P. (2011). Breast cancer growth and metastasis: Interplay between cancer stem cells, embryonic signaling pathways and epithelial-to-mesenchymal transition. Breast Cancer Res..

[32] Counter C.M., Avilion A.A., Lefeuvrel C.E., Stewart N.G., Greider C.W., Harley C.B., Bacchettil S. (1992). Telomere shortening associated with chromosome instability is arrested in immortal cells which express telomerase activity. EMBO J..

[33] Allsopp R.C., Morin G.B., DePinho R., Harley C.B., Weissman I.L. (2003). Telomerase is required to slow telomere shortening and extend replicative lifespan of HSCs during serial transplantation. Blood.

[34] Kim N.W., Piatyszek M.A., Prowse K.R., Harley C.B., West M.D., Ho P.L., Coviello G.M., Wright W.E., Weinrich S.L., Shay J.W. (1994). Specific association of human telomerase activity with immortal cells and cancer. Science.

[35] Xin Z.T., Beauchamp A.D., Calado R.T., Bradford J.W., Regal J.A., Shenoy A., Liang Y., Lansdorp P.M., Young N.S., Ly H. (2007). Functional characterization of natural telomerase mutations found in patients with hematologic disorders. Blood.

[36] Makki J., Myint O., Wynn A.A., Samsudin A.T., John D.V. (2015). Expression distribution of cancer stem cells, epithelial to mesenchymal transition, and telomerase activity in breast cancer and their association with clinicopathologic characteristics. Clin. Med. Insights Pathol..

[37] Thiery J.P., Sleeman J.P. (2006). Complex networks orchestrate epithelial–mesenchymal transitions. Nat. Rev. Mol. Cell Biol..

[38] Yang J., Mani S.A., Donaher J.L., Ramaswamy S., Itzykson R.A., Come C., Savagner P., Gitelman I., Richardson A., Weinberg R.A. (2004). Twist, a master regulator of morphogenesis, plays an essential role in tumor metastasis. Cell.

[39] Shook D., Keller R. (2003). Mechanisms, mechanics and function of epithelial-mesenchymal transitions in early development. Mech. Dev..

[40] Peinado H., Portillo F., Cano A. (2004). Transcriptional regulation of cadherins during development and carcinogenesis. Int. J. Dev. Biol..

[41] Mani S.A., Guo W., Liao M., Eaton E.N., Zhou A.Y., Brooks M., Reinhard F., Zhang C.C., Campbell L.L., Polyak K. (2008). The epithelial-mesenchymal transition generates cekks with properties of stem cells. Cell.

[42] Hollier B.G., Tinnirello A.A., Werden S.J., Evans K.W., Taube J.H., Sarkar T.R., Sphyris N., Shariati M., Kumar S.V., Battula V.L. (2013). FOXC2 expression links epithelial-mesenchymal transition and stem cell properties in breast cancer. Cancer Res..

[43] Dembinski J.L., Krauss S. (2009). Characterization and functional analysis of a slow cycling stem cell-like subpopulation in pancreas adenocarcinoma. Clin. Exp. Metastasis.

[44] Yamamoto Y., Yoshioka Y., Minoura K., Takahashi R.U., Takeshita F., Taya T., Horii R., Fukuoka Y., Kato T., Kosaka N. (2011). An integrative genomic analysis revealed the relevance of microRNA and gene expression for drug-resistance in human breast cancer cells. Mol. Cancer.

[45] Schmidt F., Efferth T. (2016). Tumor heterogeneity, single-cell sequencing, and drug resistance. Pharmaceuticals.

[46] Efferth T., Konkimalla V.B., Wang Y.F., Sauerbrey A., Meinhardt S., Zintl F., Mattern J., Volm M. (2008). Prediction of broad spectrum resistance of tumors towards anticancer drugs. Clin. Cancer Res..

[47] Longley D.B., Johnston P.G. (2005). Molecular mechanisms of drug resistance. J. Pathol..

[48] Naik P.P., Das D.N., Panda P.K., Mukhopadhyay S., Sinha N., Praharaj P.P., Agarwal R., Bhutia S.K. (2016). Implications of cancer stem cells in developing therapeutic resistance in oral cancer. Oral Oncol..

[49] Bao S., Wu Q., McLendon R.E., Hao Y., Shi Q., Hjelmeland A.B., Dewhirst M.W., Bigner D.D., Rich J.N. (2006). Glioma stem cells promote radioresistance by preferential activation of the DNA damage response. Nature.

[50] Dallas N.A., Xia L., Fan F., Gray M.J., Gaur P., van Buren G., Samuel S., Kim M.P., Lim S.J., Ellis L.M. (2009). Chemoresistant colorectal cancer cells, the cancer stem cell phenotype, and increased sensitivity to insuline-like growth factor-1 receptor inhibitor. Cancer Res..

[51] Bertolini G., Roz L., Perego P., Tortoreto M., Fontanella E., Gatti L., Pratesi G., Fabbri A., Andriani F., Tinelli S. (2009). Highly tumorigenic lung cancer CD133+ cells display stem-like features and are spared by cisplatin treatment. Proc. Natl. Acad. Sci. USA.

[52] Sahlberg S.H., Spiegelberg D., Glimelius B., Stenerlöw B., Nestor M. (2014). Evaluation of cancer stem cell markers CD133, CD44, CD24: Association with AKT isoforms and radiation resistance in colon cancer cells. PLoS ONE.

[53] Da Silva S.D., Hier M., Mlynarek A., Kowalski L.P., Alaoui-Jamali M.A. (2012). Recurrent oral cancer: Current and emerging therapeutic approaches. Front. Pharmacol..

[54] Kong Y., Peng Y., Liu Y., Xin H., Zhan X., Tan W. (2015). Twist1 and Snail link Hedgehog signaling to tumor-initiating cell-like properties and acquired chemoresistance independently of ABC transporters. Stem Cells.

[55] Sarrio D., Franklin C.K., Mackay A., Reis-Filho J.S., Isacke C.M. (2012). Epithelial and mesenchymal subpopulations within normal basal breast cell lines exhibit distinct stem cell/progenitor properties. Stem Cells.

[56] Bradford G.B., Williams B., Rossi R., Bertoncello I. (1997). Quiescence, cycling, and turnover in the primitive hematopoietic stem cell compartment. Exp. Hematol..

[57] Zhu L.-F., Hu Y., Yang C.-C., Xu X.-H., Ning T.-Y., Wang Z.-L., Ye J.-H., Liu L.-K. (2012). Snail overexpression induces an epithelial to mesenchymal transition and cancer stem cell-like properties in SCC9 cells. Lab. Investig..

[58] La Fleur L., Johansson A.C., Roberg K. (2012). A CD44high/EGFRlow subpopulation within head and neck cancer cell lines shows an epithelial-mesenchymal transition phenotype and resistance to treatment. PLoS ONE.

[59] Gottesman M.M., Pastan I. (1993). Biochemistry of multidrug resistance mediated by the multidrug transporter. Annu. Rev. Biochem..

[60] DeGorter M.K., Xia C.Q., Yang J.J., Kim R.B. (2012). Drug transporters in drug efficacy and toxicity. Annu. Rev. Pharmacol. Toxicol..

[61] Vasiliou V., Vasiliou K., Nebert D.W. (2009). Human ATP-binding cassette (ABC) transporter family. Hum. Genom..

[62] Hawley T.S., Riz I., Yang W., Wakabayashi Y., DePalma L., Chang Y.T., Peng W., Zhu J., Hawley R.G. (2013). Identification of an ABCB1 (P-glycoprotein)-positive carfilzomib-resistant myeloma subpopulation by the pluriopotent stem cell fluorescent dye Cdy1. Am. J. Hematol..

[63] Goodell M.A., Brose K., Paradis G., Conner A.S., Mulligan R.C. (1996). Isolation and functional properties of murine hematopoietic stem cells that are replicating in vivo. J. Exp. Med..

[64] Chuthapisith S., Eremin J., El-Sheemey M., Eremin O. (2010). Breast cancer chemoresistance: Emerging importance of cancer stem cells. Surg. Oncol..

[65] Scharenberg C.W., Harkey M.A., Torok-Storb B. (2002). The ABCG2 transporter is an efficient Hoechst 33342 efflux pump and is preferentially expressed by immature human hematopoietic progenitors. Blood.

[66] Simon M.C., Keith B. (2008). The role of oxygen availability in embryonic development and stem cell function. Nat. Rev. Mol. Cell Biol..

[67] Das B., Tsuchida R., Malkin D., Koren G., Baruchel S., Yeger H. (2008). Hypoxia enhances tumor stemness by increasing the invasive and tumorigenic side population fraction. Stem Cells.

[68] Fu Y., Li H., Hao X. (2017). The self-renewal signaling pathways utilized by gastric cancer stem cells. Tumor Biol..

[69] Jogi A., Ora I., Nilsson H., Lindeheim A., Makino Y., Poellinger L., Axelson H., Pahlman S. (2002). Hypoxia alters gene expression in human neuroblastoma cells toward an immature and neural crest-like phenotype. Proc. Natl. Acad. Sci. USA.

[70] Semenza G.L. (2004). Hydroxylation of HIF-1: Oxygen sensing at the molecular level. Physiology.

[71] Michiels C. (2004). Physiological and pathological responses to hypoxia. Am. J. Pathol..

[72] Liu L., Salnikov A.V., Bauer N., Aleksandrowicz E., Labsch S., Nwaeburu C., Mattern J., Gladkich J., Schemmer P., Werner J. (2014). Triptolide reverses hypoxia-induced epithelial-mesenchymal transition and stem-like features in pancreatic cancer by NF-κB downregulation. Int. J. Cancer.

[73] Majmundar A.J., Wong W.J., Simon M.C. (2010). Hypoxia-inducible factors and the response to hypoxic stress. Mol. Cell.

[74] Almog N. (2010). Molecular mechanisms underlying tumor dormancy. Cancer Lett..

[75] Yamamori T., Yasui H., Yamazumi M., Wada Y., Nakamura Y., Nakamura H., Inanami O. (2012). Ionizing radiation induces mitochondrial reactive oxygen species production accompanied by upregulation of mitochondrial electron transport chain function and mitochondrial content under control of the cell cycle checkpoint. Free Radic. Biol. Med..

[76] Wiseman H., Halliwell B. (1996). Damage to DNA by reactive oxygen and nitrogen species: Role in inflammatory disease and progression to cancer. Biochem. J..

[77] Clark D.W., Palle K. (2016). Aldehyde dehydrogenases in cancer stem cells: Potential as therapeutic targets. Ann. Transl. Med..

[78] Dontu G., Abdallah W.M., Foley J.M., Jackson K.W., Clarke M.F., Kawamura M.J., Wicha M.S. (2003). In vitro propagation and transcriptional profiling of human mammary stem/progenitor cells. Genes Dev..

[79] Diehn M., Cho R.W., Lobo N.A., Kalisky T., Dorie M.J., Kulp A.N., Qian D., Lam J.S., Ailles L.E., Wong M. (2009). Association of reactive oxygen species levels and radioresistance in cancer stem cells. Nature.

[80] Chang C.W., Chen Y.S., Chou S.H., Han C.L., Chen Y.J., Yang C.C., Huang C.Y., Lo J.F. (2014). Distinct subpopulations of head and neck cancer cells with different levels of intracellular reactive oxygen species exhibit diverse stemness, proliferation, and chemosensitivity. Cancer Res..

[81] Li X., Xu Q., Fu X., Luo W. (2014). ALDH1A1 overexpression is associated with the progression and prognosis in gastric cancer. BMC Cancer.

[82] Balicki D. (2007). Moving forward in human mammary stem cell biology and breast cancer prognostication using ALDH1. Cell Stem Cell.

[83] Huo W., Du M., Pan X., Zhu X., Li Z. (2015). Prognostic value of ALDH1 expression in lung cancer: A meta-analysis. Int. J. Clin. Exp. Med..

[84] Ikeda J., Mamat S., Tian T., Wang Y., Luo W., Rahadiani N., Aozasa K., Morii E. (2012). Reactive oxygen species and aldehyde dehydrogenase activity in Hodgkin lymphoma cells. Lab. Investig..

[85] Aponte P.M., Caicedo A. (2017). Stemness in cancer: Stem cells, cancer stem cells, and their microenvironment. Stem Cells Int..

[86] Gasparetto M., Smith C.A. (2017). ALDHs in normal and malignant hematopoietic cells: Potential new avenues for treatment of AML and other blood cancers. Chem. Biol. Interact..

[87] Tsai L.L., Yu C.C., Lo J.F., Sung W.W., Lee H., Chen S.L., Chou M.Y. (2012). Enhanced cisplatin resistance in oral-cancer stem-like cells is correlated with upregulation of excision-repair cross-complementation group 1. J. Dent. Sci..

[88] Kim N.H., Kim H.S., Li X.Y., Lee I., Choi H.S., Kang S.E., Cha S.Y., Ryu J.K., Yoon D., Fearon E.R. (2011). A p53/miRNA-34 axis regulates Snail1-dependent cancer cell epithelial-mesenchymal transition. J. Cell Biol..

[89] Wang L., Liu X., Ren Y., Zhang J., Chen J., Zhou W., Guo W., Wang X., Chen H., Li M. (2017). Cisplatin-enriching cancer stem cells confer multidrug resistance in non-small cell lung cancer via enhancing TRIB1/HDAC activity. Cell Death Dis..

[90] Croce C.M., Calin G.A. (2005). miRNAs, cancer and stem cell division. Cell.

[91] Ji Q., Hao X., Zhang M., Tang W., Meng Y., Li L., Xiang D., DeSano J.T., Bommer G.T., Fan D. (2009). MicroRNA miR-34 inhibits human pancreatic cancer tumor-initiating cells. PLoS ONE.

[92] Ma C., Ding Y.C., Yu W., Wang Q., Meng B., Huang T. (2015). MicroRNA-200c overexpression plays an inhibitory role in human pancreatic cancer stem cells by regulating epithelial-mesenchymal transition. Minerva Med..

[93] Song S.J., Ito K., Ala U., Kat L., Webster W., Sun S.M., Jongen-Lavrenic M., Manova-Todorova K., Teruya-Feldstein J., Avigan D.E. (2013). The oncogenic MicroRNA miR-22 targets the TET2 tumor suppressor to promote hematopoietic stem cell self-renewal and transformation. Cell Stem Cell.

[94] Song S.J., Polisen L., Song M.S., Ala U., Webster K., Ng C., Beringer G., Brikbak N.J., Yuan X., Cantley L.C. (2013). MicroRNA-antagonism regulates breast cancer stemness and metastasis via TET-family-dependent chromatin remodeling. Cell.

[95] De Carolis S., Bertoni S., Nati M., D’Anello L., Papi A., Tesei A., Cricca M., Bonafe M. (2016). Carbonic anhydrase 9 mRNA/microRNA34a interplay in hypoxic human mammospheres. J. Cell. Physiol..

[96] Papi A., de Carolis S., Bertoni S., Stroci G., Sceberras V., Santini D., Ceccarelli C., Taffurelli M., Orlandi M., Bonafe M. (2014). PPARγ and RXR ligands disrupt the inflammatory cross-talk in the hypoxic breast cancer stem cells niche. J. Cell. Physiol..

[97] Taylor D.D., Doellgast G.J. (1979). Quantitation of peroxidase-antibody binding to membrane fragments using column chromatography. Anal. Biochem..

[98] Bao B., Ali S., Ahmad A., Azmi A.A., Li Y., Banerjee S., Kong D., Sethi S., Sboukameel A., Padhye S.B. (2012). Hypoxia-induced aggressiveness of pancreatic cancer cells is due to increased expression of VEGF, IL-6 and miR-21, which can be attenuated by CDF treatment. PLoS ONE.

[99] Takahashi R.U., Miyazaki H., Takeshita F., Yamamoto Y., Minoura K., Ono M., Kodaira M., Tamura K., Mori M., Ochiya T. (2015). Loss of microRNA-27b contributes to breast cancer stem cell generation by activating ENPP1. Nat. Commun..

[100] Hanahan D., Weinberg R.A. (2011). Hallmarks of cancer: The next generation. Cell.

[101] Nowell P.C. (1976). The clonal evolution of tumor cell populations. Science.

[102] Gerlinger M., Rowan A.J., Horswell S., Math M., Larkin J., Endesfelder D., Gronroos E., Martinez P., Matthews N., Stewart A. (2012). Intratumor Heterogeneity and Branched Evolution Revealed by Multiregion Sequencing. N. Engl. J. Med..

[103] Martelotto L.G., Ng C.K., Piscuoglio S., Weigelt B., Reis-Filho J.S. (2014). Breast cancer intra-tumor heterogeneity. Breast Cancer Res..

[104] Clarke M.F., Fuller M. (2006). Stem cells and cancer: Two faces of eve. Cell.

[105] Li L., Xie T. (2005). Stem cell niche: Structure and function. Annu. Rev. Cell Dev. Biol..

[106] Liu H., Zhang W., Jia Y., Yu Q., Grau G.E., Peng L., Ran Y., Yang Z., Deng H., Lou J. (2013). Single-cell clones of liver cancer stem cells have the potential of differentiating into different types of tumor cells. Cell Death Dis..

[107] Ishimoto T., Sawayama H., Sugihara H., Baba H. (2014). Interaction between gastric cancer stem cells and the tumor microenvironment. J. Gastroenterol..

[108] Daverey A., Drain A.P., Kidambi S. (2015). Physical intimacy of breast cancer cells with mesenchymal stem cells elicits trastuzumab resistance through src activation. Sci. Rep..

[109] Yu Y., Xiao C.-H., Tan L.-D., Wang Q.-S., Li X.-Q., Feng Y.-M. (2014). Cancer-associated fibroblasts induce epithelial–mesenchymal transition of breast cancer cells through paracrine TGF-β signalling. Br. J. Cancer.

[110] Han M.E., Kim H.J., Shin D.H., Hwang S.H., Kang C.D., Oh S.O. (2015). Overexpression of NRG1 promotes progression of gastric cancer by regulating the self-renewal of cancer stem cells. J. Gastroenterol..

[111] Acharyya S., Oskarsson T., Vanharanta S., Malladi S., Kim J., Morris P.G., Manova-Todorova K., Leversha M., Hogg N., Seshan V.E. (2012). A CXCL1 paracrine network links cancer chemoresistance and metastasis. Cell.

[112] Tang D.G. (2012). Understanding cancer stem cell heterogeneity and plasticity. Cell Res..

[113] Hu Y., Yan C., Mu L., Huang K., Li X., Tao D., Wu Y., Qin J. (2015). Fibroblast-derived exosomes contribute to chemoresistance through priming cancer stem cells in colorectal cancer. PLoS ONE.

[114] Boelens M.C., Wu T.J., Nabet B.Y., Xu B., Qiu Y., Yoon T., Azzam D.J., Victor C.T., Wiemann B.Z., Ishwaran H. (2014). Exosome transfer from stromal to breast cancer cells regulates therapy resistance pathways. Cell.

[115] Loeffler M., Krüger J.A., Niethammer A.G., Reisfeld R.A. (2006). Targeting tumor-associated fibroblasts improves cancer chemotherapy by increasing intratumoral drug uptake. J. Clin. Investig..

[116] Liu S., Ginestier C., Ou S.J., Clouthier S.G., Patel S.H., Monville F., Korkaya H., Heath A., Dutcher J., Kleer C.G. (2012). Breast cancer stem cells are regulated by mesenchymal stem cells through cytokine networks. Cancer Res..

[117] Kalluri R., Zeisberg M. (2006). Fibroblasts in cancer. Nat. Rev. Cancer.

[118] Rafii A., Mirshahi P., Poupot M., Faussat A.M., Simon A., Ducros E., Mery E., Couderc B., Lis R., Capdet J. (2008). Oncologic trogocytosis of an original stromal cells induces chemoresistance of ovarian tumours. PLoS ONE.

[119] Frank N.Y., Schatton T., Frank M.H. (2010). The therapeutic promise of the cancer stem cell concept. J. Clin. Investig..

[120] Zhang Z., Dong Z., Lauxen I.S., Filho M.S.A., Nör J.E. (2014). Endothelial cell-secreted EGF induces epithelial to mesenchymal transition and endows head and neck cancer cells with stem-like phenotype. Cancer Res..

[121] Charles N., Ozawa T., Squatrito M., Bleau A.M., Brennan C.W., Hambardzumyan D., Holland E.C. (2013). Perivascular Nitric Oxide Activates Notch Signaling and Promotes Stem-like Character in PDGF-induced Glioma Cells. Cell Stem Cell.

[122] Lu J., Ye X., Fan F., Xia L., Bhattacharya R., Bellister S., Tozzi F., Sceusi E., Zhou Y., Tachibana I. (2013). Endothelial cells promote the colorectal cancer stem cell phenotype through a soluble form of Jagged-1. Cancer Cell.

[123] Fukumura D., Jain R.K. (2007). Tumor microenvironment abnormalities: Causes, consequences, and strategies to normalize. J. Cell. Biochem..

[124] Condeelis J., Pollard J.W. (2006). Macrophages: Obligate partners for tumor cell migration, invasion, and metastasis. Cell.

[125] Bonde A.-K., Tischler V., Kumar S., Soltermann A., Schwendener R.A. (2012). Intratumoral macrophages contribute to epithelial-mesenchymal transition in solid tumors. BMC Cancer.

[126] Korkaya H., Kim G., Davis A., Malik F., Henry N.L., Quraishi A.A., Tawakkol N., Angelo R.D., Chung S., Luther T. (2012). Activation of an IL6 inflamatory loop mediates trastuzumab resistance in HER2+ breast cancer by expanding the cancer stem cell population. Mol. Cell.

[127] Murai T. (2015). Lipid raft-mediated regulation of hyaluronan-CD44 interactions in inflammation and cancer. Front. Immunol..

[128] Oskarsson T., Acharyya S., Zhang X.H.-F., Vanharanta S., Tavazoie S.F., Morris P.G., Downey R.J., Manova-Todorova K., Brogi E., Massagué J. (2011). Breast cancer cells produce tenascin C as a metastatic niche component to colonize the lungs. Nat. Med..

[129] Ostman A., Augsten M. (2009). Cancer-associated fibroblasts and tumor growth-bystanders turning into key players. Curr. Opin. Genet. Dev..

[130] Dvorak H.F. (1986). Tumors: Wounds that do not heal. Similarities between tumor stroma generation and wound healing. N. Engl. J. Med..

[131] Mueller M.M., Fusenig N.E. (2004). Friends or foes—Bipolar effects of the tumour stroma in cancer. Nat. Rev. Cancer.

[132] Orimo A., Gupta P.B., Sgroi D.C., Arenzana-Seisdedos F., Delaunay T., Naeem R., Carey V.J., Richardson A.L., Weinberg R.A. (2005). Stromal fibroblasts present in invasive human breast carcinomas promote tumor growth and angiogenesis through elevated SDF-1/CXCL12 secretion. Cell.

[133] Gabbiani G., Ryan G.B., Majne G. (1971). Presence of modified fibroblasts in granulation tissue and their possible role in wound contraction. Experientia.

[134] Martinez-Outschoorn U.E., Goldberg A., Lin Z., Ko Y.H., Flomenberg N., Wang C., Pavlides S., Pestell R.G., Howell A., Sotgia F. (2011). Anti-estrogen resistance in breast cancer is induced by the tumor microenvironment and be overcome by inhibiting mitochondrial function in epithelial cancer cells. Cancer Biol. Ther..

[135] Calabrese C., Poppleton H., Kocak M., Hogg T.L., Fuller C., Hamner B., Oh E.Y., Gaber M.W., Finklestein D., Allen M. (2007). A Perivascular niche for brain tumor stem cells. Cancer Cell.

[136] Krishnamurthy S., Dong Z., Vodopyanov D., Imai A., Joseph I., Prince M.E., Wicha M.S., Nör J.E. (2010). Endothelial cell-initiated signaling promotes the survival and self-renewal of cancre stem cells. Cancer Res..

[137] Shibue T., Weinberg R.A. (2017). EMT, CSCs, and drug resistance: The mechanistic link and clinical implications. Nat. Rev. Clin. Oncol..

[138] Lewis C.E., Pollard J.W. (2006). Distinct role of macrophages in different tumor microenvironments. Cancer Res..

[139] Wong G.S., Rustgi A.K. (2013). Matricellular proteins: Priming the tumour microenvironment for cancer development and metastasis. Br. J. Cancer.

[140] Shao Z.M., Nguyen M., Barsky S.H. (2000). Human breast carcinoma desmoplasia is PDGF initiated. Oncogene.

[141] Giannoni E., Bianchini F., Masieri L., Serni S., Torre E., Calorini L., Chiarugi P. (2010). Reciprocal activation of prostate cancer cells and cancer-associated fibroblasts stimulates epithelial-mesenchymal transition and cancer stemness. Cancer Res..

[142] Hugo H.J., Lebret S., Tomaskovic-Crook E., Ahmed N., Blick T., Newgreen D.F., Thompson E.W., Ackland M.L. (2012). Contribution of fibroblast and mast cell (afferent) and tumor (efferent) IL-6 effects within the tumor microenvironment. Cancer Microenviron..

[143] Tsuyada A., Chow A., Wu J., Somlo G., Chu P., Loera S., Luu T., Li A.X., Wu X., Ye W. (2012). CCL2 mediates cross-talk between cancer cells and stromal fibroblasts that regulates breast cancer stem cells. Cancer Res..

[144] Valenti G., Quinn H.M., Heynen G.J.J.E., Lan L., Holland J.D., Vogel R., Wulf-Goldenberg A., Birchmeier W. (2017). Cancer stem cells regulate cancer-associated fibroblasts via activation of hedgehog signaling in mammary gland tumors. Cancer Res..

[145] Wang R., Chadalavada K., Wilshire J., Kowalik U., Hovinga K.E., Geber A., Fligelman B., Leversha M., Brennan C., Tabar V. (2010). Glioblastoma stem-like cells give rise to tumour endothelium. Nature.

[146] Medici D., Shore E.M., Lounev V.Y., Kaplan F.S., Kalluri R., Olsen B.R. (2010). Conversion of vascular endothelial cells into multipotent stem-like cells. Nat. Med..

[147] Carmeliet P. (2000). Mechanisms of angiogenesis and arteriogenesis. Nat. Med..

[148] Ricci-Vitiani L., Pallini R., Biffoni M., Todaro M., Invernici G., Cenci T., Maira G., Parati E.A., Stassi G., Larocca L.M. (2010). Tumour vascularization via endothelial differentiation of glioblastoma stem-like cells. Nature.

[149] Ponti D., Costa A., Zaffaroni N., Pratesi G., Petrangolini G., Coradini D., Pilotti S., Pierotti M.A., Daidone M.G. (2005). Isolation and in vitro propagation of tumorigenic breast cancer cells with stem/progenitor cell properties. Cancer Res..

[150] Zhao Y., Dong J., Huang Q., Lou M., Wang A., Lan Q. (2010). Endothelial cell transdifferentiation of human glioma stem progenitor cells in vitro. Brain Res. Bull..

[151] Bussolati B., Grange C., Sapino A., Camussi G. (2009). Endothelial cell differentiation of human breast tumour stem/progenitor cells. J. Cell. Mol. Med..

[152] Sparmann A., Bar-Sagi D. (2004). Ras-induced interleukin-8 expression plays a critical role in tumor growth and angiogenesis. Cancer Cell.

[153] Lu H., Ckauser K.R., Tam W.L., Frose J., Ye X., Eaton E.N., Reinhardt F., Donnenberg V.S., Bhargava R., Carr S.A. (2014). A breast cancer stem cell niche supported by juxtacrine signalling from monocytes and macrophages. Nat. Cell Biol..

[154] Qian B.Z., Li J., Zhang H., Kitamura T., Zhang J., Campion L.R., Kaiser E.A., Snyder L.A., Pollard J.W. (2011). CCL2 recruits inflammtatory monocytes to facilitate breast tumour metastasis. Nature.

[155] Robinson S.C., Scott K.A., Wilson J.L., Thompson R.G., Proudfoot A.E., Balkwill F.R. (2003). A chemokine receptor antagonist inhibits experimental breast tumor growth. Cancer Res..

[156] Botelho M., Alves H. (2016). Significance of Cancer Stem Cells in Anti-Cancer Therapies. Int. J. Immunother. Cancer Res..

[157] Plaks V., Kong N., Werb Z. (2015). The cancer stem cell niche: How essential is the niche in regulating stemness of tumor cells?. Cell Stem Cell.

[158] Sekulic A., Migden M.R., Oro A.E., Dirix L., Lewis K.D., Hainsworth J.D., Solomon J.A., Yoo S., Arron S.T., Friedlander P.A. (2012). Efficacy and safety of vismodegib in advanced basal-cell carcinoma. N. Engl. J. Med..

[159] Sekulic A., Migden M.R., Basset-Seguin N., Garbe C., Gesierich A., Lao C.D., Miller C., Mortier L., Murrell D.F., Hamid O. (2017). Long-term safety and efficacy of vismodegib in patients with advanced basal cell carcinoma: Final update (30-month) of the pivotal ERIVANCE BCC study. BMC Cancer.

[160] Schulze B., Meissner M., Ghanaati S., Burck I., Rödel C., Balermpas P. (2016). Hedgehog pathway inhibitor in combination with radiation therapy for basal cell carcinomas of the head and neck. Strahlenther. Onkol..

[161] Kaiser J. (2015). The cancer stem cell gamble. Science.

[162] Gupta P.B., Onder T.T., Jiang G., Tao K., Kuperwasser C., Weinberg R.A., Lander E.S. (2009). Identification of selective inhibitors of cancer stem cells by high-throughput screening. Cell.

[163] Mai T.T., Hamai A., Hienzsch A., Cañeque T., Muller S., Wicinski J., Cabaud O., Leroy C., David A., Acevedo V. (2017). Salinomycin kills cancer stem cells by sequestering iron in lysosomes. Nat. Chem..

[164] Tallman M.S., Altman J.K. (2009). How I treat acute promyelocytic leukemia. Leukemia.

[165] Yan Y., Li Z., Xu X., Chen C., Wei W., Fan M., Chen X., Li J.J., Wang Y., Huang J. (2016). All-trans retinoic acids induce differentiation and sensitize a radioresistant breast cancer cells to chemotherapy. BMC Complement. Altern. Med..

[166] Hirsch H.A., Iliopoulos D., Tsichlis P.N., Struhl K. (2009). Metformin selectively targets cancer stem cells, and acts together with chemotherapy to block tumor growth and prolong remission. Cancer Res..

[167] Del Barco S., Vazquez-Martin A., Cufí S., Oliveras-Ferraros C., Bosch-Barrera J., Joven J., Martin-Castillo B., Menendez J.A. (2011). Metformin: Multi-faceted protection against cancer. Oncotarget.

[168] Sonnenblick A., Agbor-Tarh D., Bradbury I., di Cosimo S., Azim H.A., Fumagalli D., Sarp S., Wolff A.C., Andersson M., Kroep J. (2017). Impact of diabetes, insulin, and metformin use on the outcome of patients with human epidermal growth factor receptor 2-positive primary breast cancer: Analysis from the ALTTO phase III randomized trial. J. Clin. Oncol..

[169] Son K., Fujioka S., Iida T., Furukawa K., Fujita T., Yamada H., Chiao P.J., Yanaga K. (2009). Doxycycline induces apoptosis in PANC-1 pancreatic cancer cells. Anticancer Res..

[170] Duivenvoorden W.C., Popovic S.V., Lhotak S., Seidlitz E., Hirte H.W., Tozer R.G., Singh G. (2002). Doxyxycle decreases tumor burden in bone metastasis model of human breast cancer. Cancer Res..

[171] De Francisco E.M., Maggiolini M., Tanowitz H.B., Sotgia F., Lisanti M.P. (2017). Targeting hypoxic cancer stem cells (CSCs) with Doxyxyxline: Implications for optimizing anti-angiogwnic therapy. Oncotarget.

[172] Raha D., Wilson T.R., Peng J., Peterson D., Yue P., Evangelista M., Wilson C., Merchant M., Settleman J. (2014). The cancer stem cell marker aldehyde dehydrogenase is required to maintain a drug-tolerant tumor cell subpopulation. Cancer Res..

[173] Moreb J.S., Muhoczy D., Ostmark B., Zucali J.R. (2007). RNAi-mediated knockdown of aldehyde dehydrogenase class-1A1 and class-3A1 is specific and reveals that each contributes equally to the resistance against 4-hydroperoxycyclophosphamide. Cancer Chemother. Pharmacol..

[174] Kummar S., Kinders R., Rubinstein L., Parchment R.E., Murgo A.J., Collins J., Pickeral O., Low J., Steinberg S.M., Gutierrez M. (2007). Compressing drug development timelines in oncology using phase “0” trials. Nat. Rev. Cancer.

[175] Smalley W.E., DuBois R.N. (1997). Colorectal cancer and nonsteroidal anti-inflammatory drugs. Adv. Pharmacol..

[176] Valverde A., Peñarando J., Cañas A., López-Sánchez L.M., Conde F., Hernández V., Peralbo E., López-Pedrera C., de la Haba-Rodríguez J., Aranda E. (2015). Simultaneous inhibition of EGFR/VEGFR and cyclooxygenase-2 targets stemness-related pathways in colorectal cancer cells. PLoS ONE.

[177] Provenzano P.P., Cuevas C., Chang A.E., Goel V.K., VonHoff D.D., Hingorani S.R. (2012). Enzymatic targeting of the stroma ablates physical barriers to treatment of pancreatic ductal adenocarcinoma. Cancer Cell.

